# Supplementary material for: DMRforPairs: identifying Differentially Methylated Regions between unique samples using array based methylation profiles
Source: BMC Bioinformatics. 2014 May 15;15:141. doi: 10.1186/1471-2105-15-141 (PMC4046028; doi:10.1186/1471-2105-15-141)
Supplement: Additional file 2 — DMRforPairs output for the comparison of A431-MCF7 and NA17018-NA17105. Please start from the HTML files in each folder. Available via the BMC Bioinformatics website. [file 1471-2105-15-141-S2.zip › 1394847754114233_MOESM2_ESM/A431_MCF7/figures/39040.pdf]

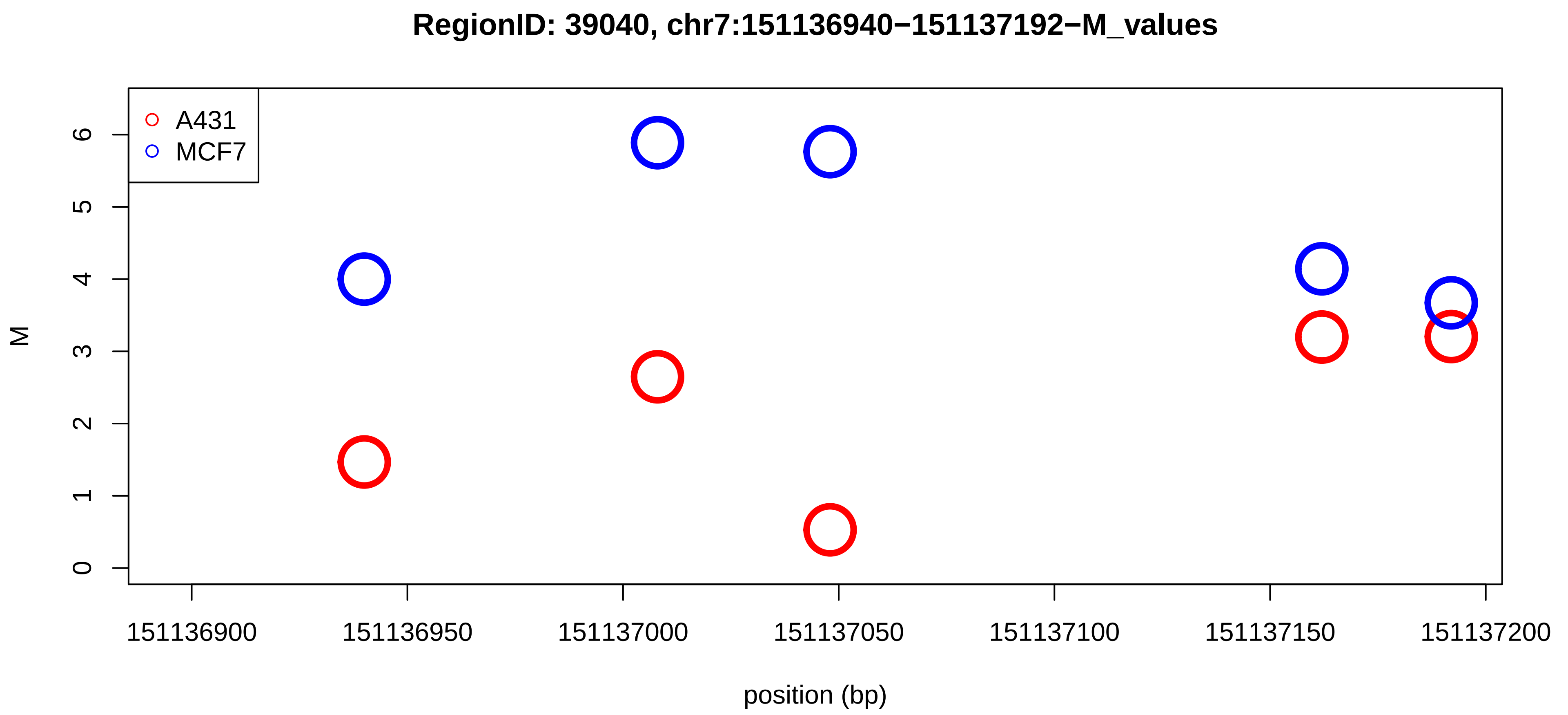
RegionID: 39040, chr7:151136940−151137192−M_values
A431
MCF7
M
0
1
2
3
4
5
6
151136900
151136950
151137000
151137050
151137100
151137150
151137200
position (bp)

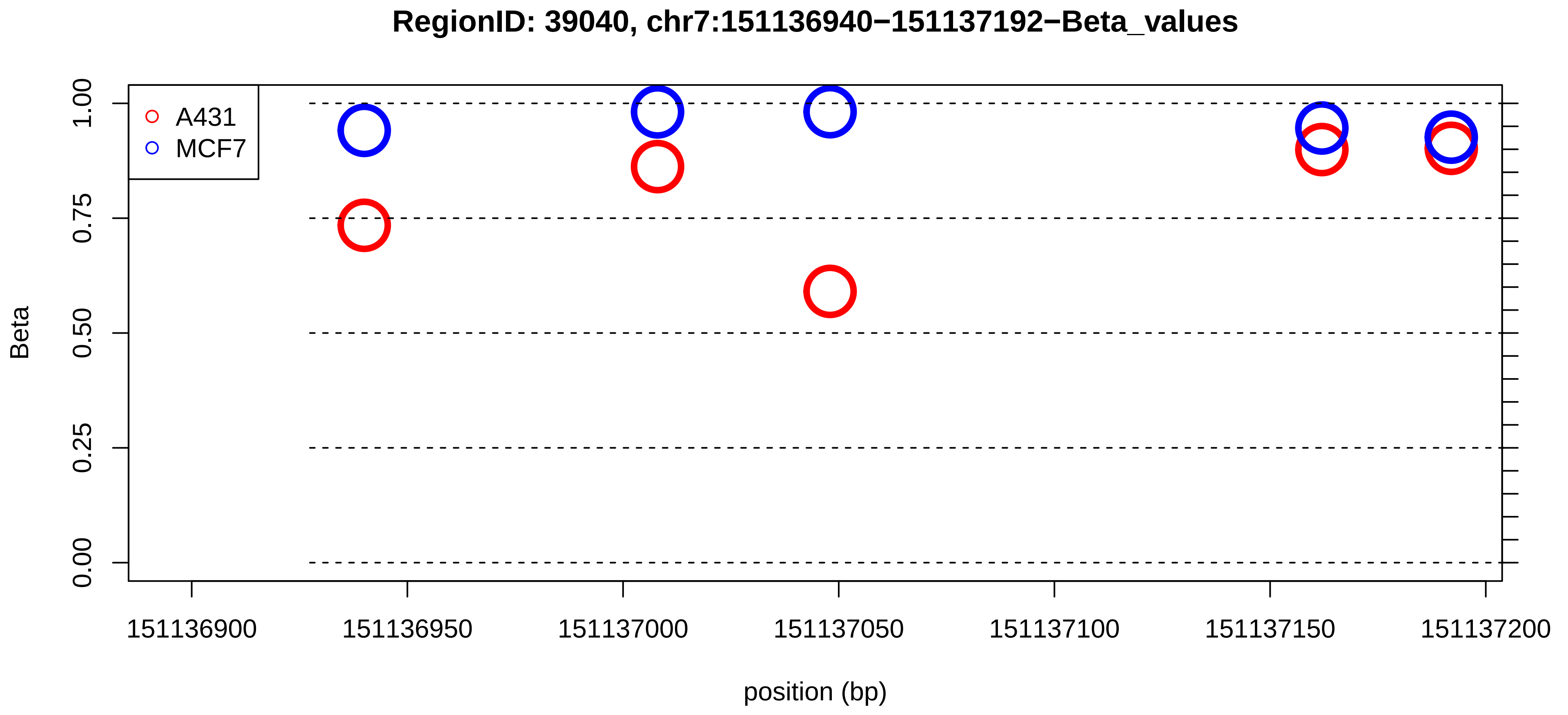
RegionID: 39040, chr7:151136940−151137192−Beta_values
A431
MCF7
Beta
0.00
0.25
0.50
0.75
1.00
151136900
151136950
151137000
151137050
151137100
151137150
151137200
position (bp)

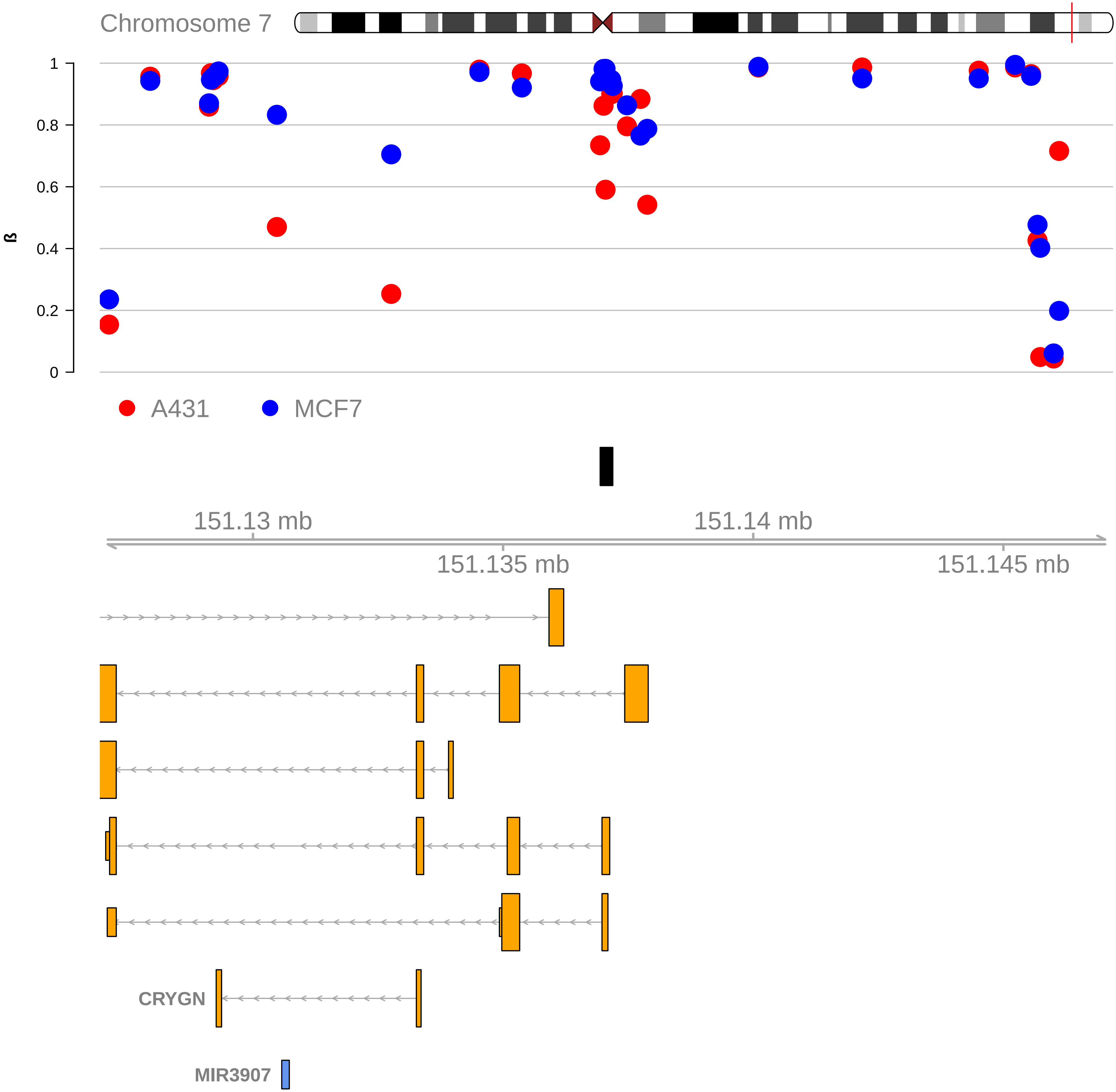

Chromosome 7
1
0.8
0.6
0.4
0.2
0
ß
A431
MCF7
151.13 mb
151.14 mb
151.135 mb
151.145 mb
CRYGN
MIR3907
